# Impact of diagnosis-to-treatment interval on mortality in patients with early-stage breast cancer: a retrospective nationwide Korean cohort

**DOI:** 10.1186/s12905-025-03780-6

**Published:** 2025-05-22

**Authors:** Sung Hoon Jeong, Seong Min Chun, Hyunji Lee, Miji Kim, Ja-Ho Leigh

**Affiliations:** 1 Department of Health Policy and Management, Inje University, 50834 Gimhae, Republic of Korea; 2 National Traffic Injury Rehabilitation Research Institute, National Traffic Injury Rehabilitation Hospital, 12564, Yangpyeong, Republic of Korea; 3 Department of Rehabilitation Medicine, Seoul National University Hospital, 03080, Seoul, Republic of Korea; 4 Department of Physical Medicine and Rehabilitation, Soonchunhyang University Hospital Seoul, Soonchunhyang University College of Medicine, 04401, Seoul, Republic of Korea; 5 Department of Rehabilitation Medicine, National Traffic Injury Rehabilitation Hospital, 12564 Yangpyeong, Republic of Korea; 6 Department of Biostatistics and Computing, Graduate School of Yonsei University, 03722 Seoul, Republic of Korea; 7 Institute of Health Policy and Management, Medical Research Center, Seoul National University, 03080, Seoul, Republic of Korea

**Keywords:** Breast surgery, Oncology, Risk factor, 5-year mortality, Time-to-treatment, Epidemiology, Early-stage

## Abstract

**Background:**

The diagnosis-to-first-treatment interval (DFTI) is an important prognostic factor and a major concern for patients with breast cancer as well as their clinicians. It may be particularly important for patients with early-stage breast cancer. The aim of this study was to investigate the association between DFTI and risk of mortality in patients with new-onset early-stage breast cancer.

**Methods:**

This nationwide, retrospective cohort study utilized data from the Korean National Health Insurance database and the Korea National Cancer Incidence Database (2006–2017). By using 1:5 propensity score matching, 3,625 participants with a DFTI < 60 days and 725 with a DFTI ≥ 60 days were included in the analysis. Cox proportional hazard regression models were used to examine the association between the DFTI and 5-year all-cause mortality risk.

**Results:**

Compared with patients with breast cancer with a DFTI < 60 days, patients with a DFTI ≥ 60 days had a higher 5-year mortality risk (hazard ratio [95% confidence interval], 2.09 [1.43–3.06]). Similarly, sensitivity analysis with a 45-day threshold revealed higher mortality in patients with a DFTI ≥ 45 days (HR [95% CI], 1.49 [1.14–1.96]) than their counterparts with a DFTI < 45 days. This association was greater for patients with low household income, those who lived in rural areas, and those with a high Charlson comorbidity index.

**Conclusions:**

A DFTI ≥ 60 days was associated with mortality risk in patients with early-stage breast cancer. These results emphasize the importance of closely monitoring the waiting times of this patients population and ensuring timely treatment.

**Supplementary Information:**

The online version contains supplementary material available at 10.1186/s12905-025-03780-6.

## Background

Breast cancer is among the most frequently diagnosed cancer types worldwide [1], leading to a significant number of cancer-related deaths among women. Breast cancer accounts for 24.2% of total cancer incidence and 15.5% of cancer-related deaths [2]. Although breast cancer exhibits relatively high survival rates compared with other cancers, survivors frequently face long-term consequences related to the disease and its treatment, thereby affecting their quality of life and overall well-being [3].

This renders breast cancer as a major public health concern, conferring the highest cancer disability-adjusted life expectancy [2, 4]. In South Korea, the incidence of breast cancer is expected to increase rapidly due to the adoption of a Westernized lifestyle [5]. The incidences of other common cancers, such as stomach, colon, and lung cancer, have decreased, while that of breast cancer steadily increased since 1999, prompting interest in breast cancer prognosis [6].

Among various prognostic factors, the diagnosis-to-first-treatment interval (DFTI) is an important determinant of mortality in patients with breast cancer and is a key concern for patients and clinicians [7–9]. Following a breast cancer diagnosis, patients are concerned about the potential impact of delayed treatment on metastasis and mortality [10]. According to the National Breast and Cervical Cancer Early Detection Program, the DFTI for patients with breast cancer should not exceed 60 days [11]. The time to surgery is a modifiable factor, dependent on clinical decision-making discretion; therefore, it holds clinical importance. Thus, the timeliness of treatment has been proposed as a quality-control and assurance parameter in the context of breast cancer care [12]. Delays in surgical treatment can worsen patients’ anxiety and contribute to adverse effects, such as disease progression or further delays in adjuvant treatment [10, 13–15]. Moreover, most patients fear that cancer may progress during the DFTI, which potentially can cause concern for the treating physician [16]. In Korea, disparities in the distribution of medical facilities and specialists between urban and rural areas, along with the centralization of healthcare services in Seoul, has increased treatment demand in metropolitan areas, thereby prolonging waiting times [17, 18]. This structural imbalance in healthcare resources exacerbates delays in treatment, particularly for patients who require specialized care in high-demand regions [18]. In this context, the effect of the DFTI becomes even more critical as it can vary depending on the stage of cancer [19]. Patients with early-stage cancer typically exhibit a lower baseline mortality rate and are more vulnerable to the adverse effects of delayed treatment compared to those with late-stage disease [10, 20]. Historical concerns regarding the impact of delayed breast cancer treatment date back several decades, with foundational studies emphasizing the importance of timely intervention to improve survival outcomes [21]. Therefore, timely treatment is more emphasized for patients with early-stage breast cancer.

Recent studies have reported conflicting results regarding the effect of the DFTI on mortality. Although some studies suggest that a shorter DFTI may positively affect disease progression, mortality rate, and quality of life [19, 22, 23], others suggest that DFTI has no significant effect on patient mortality [24–26]. In particular, most studies conducted in Korea have utilized single-center cohorts, resulting in inconsistent findings [26, 27]. Moreover, while certain studies suggest that longer DFTI worsens overall survival in patients with early-stage breast cancer, others report no significant effect on survival, even among early-stage cases [26, 28]. Therefore, we conducted this nationwide population-based matched cohort study to comprehensively evaluate the risk of all-cause mortality, based on the DFTI, among the entire breast cancer population in Korea.

## Methods

### Study design

This retrospective cohort study included patients with breast cancer diagnosed between 2008 and 2015, and their survival was tracked until 2017. The index date of follow-up was the date of diagnosis of new-onset breast cancer, whereas the end date was the date of loss to follow-up, death, or the end of the study period (December 31, 2017), whichever occurred first [29].

### Setting and data source

This nationwide, population-based cohort study included all adult patients diagnosed with breast cancer in South Korea between 2006 and 2017, based on data from the National Health Insurance Service (NHIS) and the Korea National Cancer Incidence Database (KNCI DB).

South Korea’s single-payer universal health coverage system ensures that > 99% of the population is covered by the NHIS, thereby minimizing selection bias and providing comprehensive population-level data. The NHIS database includes detailed information on diagnoses, treatments, surgical history, prescriptions, demographics, socioeconomic status, and geographic data [30]. Previous studies have reported that the NHIS data demonstrate a sensitivity of 98.1% for breast cancer [31], confirming the high accuracy and completeness of the data. The KNCI DB, managed by the Korea Central Cancer Registry (KCCR), is a national population-based database that includes comprehensive information on all cancer diagnoses in Korea, such as nationwide aggregated cancer cases, patient age, sex, date and location of primary cancer diagnosis, and staging information, including SEER stages [6]. We linked the SEER stage data from the KNCI DB with the NHIS data to obtain information on early-stage cancers (i.e., localized stage), which is a method previously validated in studies for its reliability and completeness [32].

All data are available in the database of the Korean National Health Insurance Sharing Service (https://nhiss.nhis.or.kr) and can be accessed upon reasonable request. The study protocol was approved by the Institutional Review Board of Seoul National University Hospital (approval no: E-2206–076–1332; Seoul, Republic of Korea). Owing to the retrospective nature of the study, the requirement for informed consent was waived.

### Study population

We collected the data of 161,514 adults with breast cancer from a historical cohort enrolled between January 1, 2006 and December 31, 2017 by integrating the NHIS and KNCI DB. All patients had the International Statistical Classification of Diseases and Related Health Problems, Tenth Revision (ICD-10) code C50 for breast cancer.

Our study population focused on patients with early-stage breast cancer as defined based on SEER staging [33, 34]. The SEER stage at diagnosis was classified as localized (i.e., invasive cancer limited to the organ of origin; code 1), regional (i.e., tumor extension beyond the limits of organ of origin; code 2–4), distant disease (i.e., spread to distant areas from the primary tumor; code 7), and unknown [35, 36]. Therefore, only patients with localized SEER stages suitable for our study purposes were included. A two-year washout period (2006–2007) was applied to identify patients with new-onset breast cancer. On account, our dataset begins in 2006, this washout period was implemented as a stricter criterion to exclude individuals who may have been diagnosed before 2006, thereby minimizing bias from pre-existing cases [31]. Furthermore, this study included treatment type as a covariate in analyzing the relationship between DFTI and mortality. Treatment type was categorized based on the treatment received during the 1-year follow-up period following a new-onset breast cancer diagnosis. On account, our data are available only through December 31, 2017; patients diagnosed with breast cancer in 2016 and 2017 were excluded from the analysis to ensure a sufficient follow-up period for categorizing treatment type and subsequently assessing mortality. Patients diagnosed with cancers other than breast cancer within the 5 years before the breast cancer diagnosis [19], and those who underwent surgical treatment (ICD-10: N7130-N7135) before their breast cancer diagnosis were excluded.

To reduce heterogeneity between the clinical states of patients, only those who received surgical treatment for breast cancer (ICD-10: N7130-N7135) within 1 year of the first diagnosis were included in this study [37]. Additionally, patients who died after the first diagnosis but before treatment were excluded to avoid bias in time-to-event outcomes [38, 39]. These patients were all limited to individuals who received initial treatment at hospital, general hospital, or tertiary hospitals. Finally, all male patients and female patients younger than 20 years were excluded, as were those whose data regarding independent variables were missing. After applying the exclusion criteria, a total of 59,430 patients were included in this study. Among them, 725 patients were categorized into the ≥ 60 days DFTI group, while 58,705 patients were classified into the < 60 days DFTI group.

**Fig. 1:**
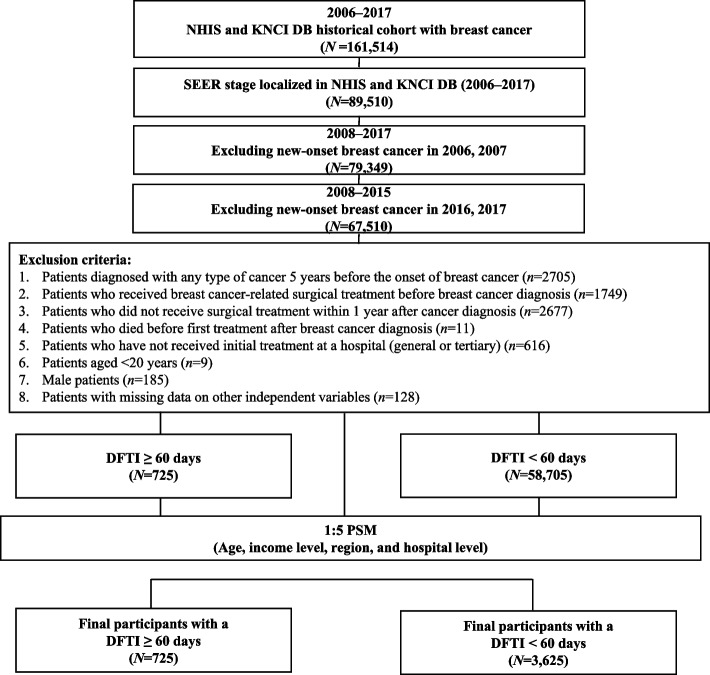
The study participant selection process

We matched participants in the ≥ 60 days with those in the < 60 days DFTI group using propensity score matching (PSM). The propensity score was derived using logistic regression to calculate the probability of treatment within 60 days, with covariates of age, income level, region, and healthcare institution type. After calculating the propensity score, we performed 1:5 greedy matching using the OneToManyMTCH macro for SAS (SAS Institute Inc., Cary, NC, USA) (Fig. 1) [40].

### Study variables

The outcome variable was 5-year all-cause mortality. The survival period of patients with early-stage breast cancer was defined as the period from the index date (the date of new-onset breast cancer diagnosis) to the end date (i.e., death or censoring). The index time refers to the day the patient was first diagnosed with new-onset breast cancer [19, 37]. Individuals were observed for up to 5 years, and “death” was assigned as the outcome for individuals who died within 5 years of their first diagnosis.

The primary variable of interest was the DFTI, defined as the interval between the date of first diagnosis and the date of first surgery [37]. Treatment delays of ≥ 60 days affect prognosis [10, 11, 20]; therefore, closely observing the patient’s condition on day 60 after the postponement of surgery is necessary. Accordingly, we subdivided the DFTI group into two subgroups based on the 60-day cutoff (i.e., ≥ 60 days and < 60 days).

Potential confounding factors included variables that could affect mortality and the DFTI. These variables included age (20–54 years, 55–64 years, or ≥ 65 years); residential area (i.e., urban, suburban, or rural); income level (i.e., low, mid-low, mid-high, or high); disability status (i.e., yes or no); healthcare institution type (i.e., hospital, general hospital, or tertiary hospital); Charlson comorbidity index (CCI); and type of treatment. The CCI is used to evaluate patients’ comorbidities that may alter mortality risk and is used in longitudinal studies [41]. The CCI was calculated using the ICD-10 score for each comorbidity by weighting 1–6 points for 19 comorbidities under the following categories: myocardial, vascular, lung, endocrine, kidney, gastrointestinal, cancer/immune, and neurological comorbidities [41, 42]. Participants were divided into three groups, based on their CCI score: 0, 1, and ≥ 2. Household income levels were divided into quartiles based on household insurance premiums. Disability status was classified into 15 types based on the disability rating standards set by Korea's Ministry of Health and Welfare, as well as through a specialist’s diagnosis [43, 44]. The patient's place of residence was categorized based on the administrative district, which comprised capital cities, metropolitan areas, and provinces. Urban areas included cities within the administrative districts of the capital city and metropolitan areas, typically with a population of approximately one-half a million residents. Suburban areas included cities within administrative districts of provinces, with populations ranging from < 50,000 to 500,000 residents. Rural areas included within provincial administrative districts are generally inhabited by < 50,000 residents [45]. Patients were also classified based on the type of treatment they received within 1 year of diagnosis.

### Statistical analysis

Patients with early-stage breast cancer were not randomly assigned to groups based on DFTI at baseline; thus, 1:5 PSM was utilized to minimize selection bias. To ascertain whether the two groups divided based on the DFTI at baseline (60-day cutoff) exhibited similar characteristics, the basic attributes of the groups were compared using Chi-square tests. Kaplan–Meier survival curves and log-rank tests were used to compare the survival rates based on the DFTI (≥ 60 days and < 60 days) groups. A multivariate-adjusted Cox proportional hazards model was used to examine the effect of the DFTI on 5-year mortality [46]. Additionally, based on recent studies conducted on the Korean population, we conducted a sensitivity analysis using a DFTI threshold of 45 days [47]. All Cox proportional hazards models were fully adjusted for the covariates presented in Table 1. Effect sizes were expressed as hazard ratios (HRs) and 95% confidence intervals (CIs). Furthermore, subgroup analysis through stratified analysis was performed to investigate the relationship between the DFTI and mortality based on household income level, residential area, disability, and CCI. Statistical significance was set at *p* < 0.05. All data analyses were performed using SAS 9.4 (SAS Institute Inc., Cary, NC, USA) and R (ver 4.3.2; R Foundation, Vienna, Austria) programs.

**Table 1:** Baseline characteristics of the DFTI groups before and after PSM

Variable	Before PSM							After PSM						
	**Total**		**DFTI**				***p*-value**	**Total**		**DFTI**				***p*-value**
			** ≥ 60 days**		** < 60 days**					** ≥ 60 days**		** < 60 days**		
**Total**	**n**	**(%)**	**n**	**(%)**	**n**	**(%)**		**n**	**(%)**	**n**	**(%)**	**n**	**(%)**	
	**59,430**	**(100.0)**	**725**	**(1.2)**	**58,705**	**(98.8)**		**4350**	**(100.0)**	**725**	**(16.7)**	**3625**	**(83.3)**	
**Age (y)**							<.0001							1.0000
20–54	3,8897	(65.5)	421	(1.1)	38,476	(98.9)		2526	(58.1)	421	(16.7)	2105	(83.3)	
55–64	12,546	(21.1)	163	(1.3)	12,383	(98.7)		978	(22.5)	163	(16.7)	815	(83.3)	
≥ 65	7,987	(13.4)	141	(1.8)	7846	(98.2)		846	(19.4)	141	(16.7)	705	(83.3)	
**Residential area**							0.0636							1.0000
Urban	28,499	(48.0)	376	(1.3)	28,123	(98.7)		2256	(51.9)	376	(16.7)	1880	(83.3)	
Suburban	15,576	(26.2)	166	(1.1)	15,410	(98.9)		996	(22.9)	166	(16.7)	830	(83.3)	
Rural	15,355	(25.8)	183	(1.2)	15,172	(98.8)		1098	(25.2)	183	(16.7)	915	(83.3)	
**Household income level**							0.2668							1.0000
Low	13,935	(23.4)	181	(1.3)	13,754	(98.7)		1086	(25.0)	181	(16.7)	905	(83.3)	
Mid-low	10,582	(17.8)	137	(1.3)	10,445	(98.7)		822	(18.9)	137	(16.7)	685	(83.3)	
Mid-high	13,491	(22.7)	171	(1.3)	13,320	(98.7)		1026	(23.6)	171	(16.7)	855	(83.3)	
High	21,422	(36.0)	236	(1.1)	21,186	(98.9)		1416	(32.6)	236	(16.7)	1180	(83.3)	
**Healthcare institution type**							<.0001							1.0000
Hospital	1,582	(2.7)	8	(0.5)	1574	(99.5)		48	(1.1)	8	(16.7)	40	(83.3)	
General hospital	16,004	(26.9)	115	(0.7)	15,889	(99.3)		690	(15.9)	115	(16.7)	575	(83.3)	
Tertiary hospital	41,844	(70.4)	602	(1.4)	41,242	(98.6)		3612	(83.0)	602	(16.7)	3010	(83.3)	
**Disability**							0.0002							0.0378
No	56,608	(95.3)	669	(1.2)	55,939	(98.8)		4087	(94.0)	669	(16.4)	3418	(83.6)	
Yes	2822	(4.7)	56	(2.0)	2766	(98.0)		263	(6.0)	56	(21.3)	207	(78.7)	
**CCI**							0.5045							0.3625
0	21,941	(36.9)	280	(1.3)	21,661	(98.7)		1582	(36.4)	280	(17.7)	1302	(82.3)	
1	17,874	(30.1)	205	(1.1)	17,669	(98.9)		1254	(28.8)	205	(16.3)	1049	(83.7)	
≥ 2	19,615	(33.0)	240	(1.2)	19,375	(98.8)		1514	(34.8)	240	(15.9)	1274	(84.1)	
**Treatment type**							<.0001							<.0001
OP	1,941	(3.3)	72	(3.7)	1869	(96.3)		187	(4.3)	72	(38.5)	115	(61.5)	
OP + CHEMO	15,553	(26.2)	269	(1.7)	15,284	(98.3)		1210	(27.8)	269	(22.2)	941	(77.8)	
OP + RADIO	1,430	(2.4)	63	(4.4)	1367	(95.6)		167	(3.8)	63	(37.7)	104	(62.3)	
OP + CHEMO + RADIO	40,506	(68.1)	321	(0.8)	40,185	(99.2)		2786	(64.1)	321	(11.5)	2465	(88.5)	

*PSM* Propensity score matching, *DFTI* Diagnosis-to-first-treatment interval, *CCI* Charlson comorbidity index, *OP* Operation, *CHEMO* Chemotherapy, *RADIO* Radiotherapy

## Results

Table 1 presents the baseline characteristics of the study population before and after PSM. After PSM, 3,625 patients with a DFTI of < 60 days and 725 patients with a DFTI of ≥ 60 days were included. Following PSM, no significant differences were observed between the two groups in terms of age, residential area, household income level, type of healthcare institution, or CCI.

Moreover, among the total 4350 patients, 131 (3.0%) died during the study period. In particular, 87 (2.4%) deaths occurred in the DFTI < 60 days group, whereas 44 (6.1%) deaths occurred in the DFTI ≥ 60 days group. Univariate analysis identified significant differences in the risk of all-cause mortality across groups stratified by DFTI, age, residential area, presence of disability, CCI, and type of treatment received (Supplementary Table 1).

**Fig. 2:**
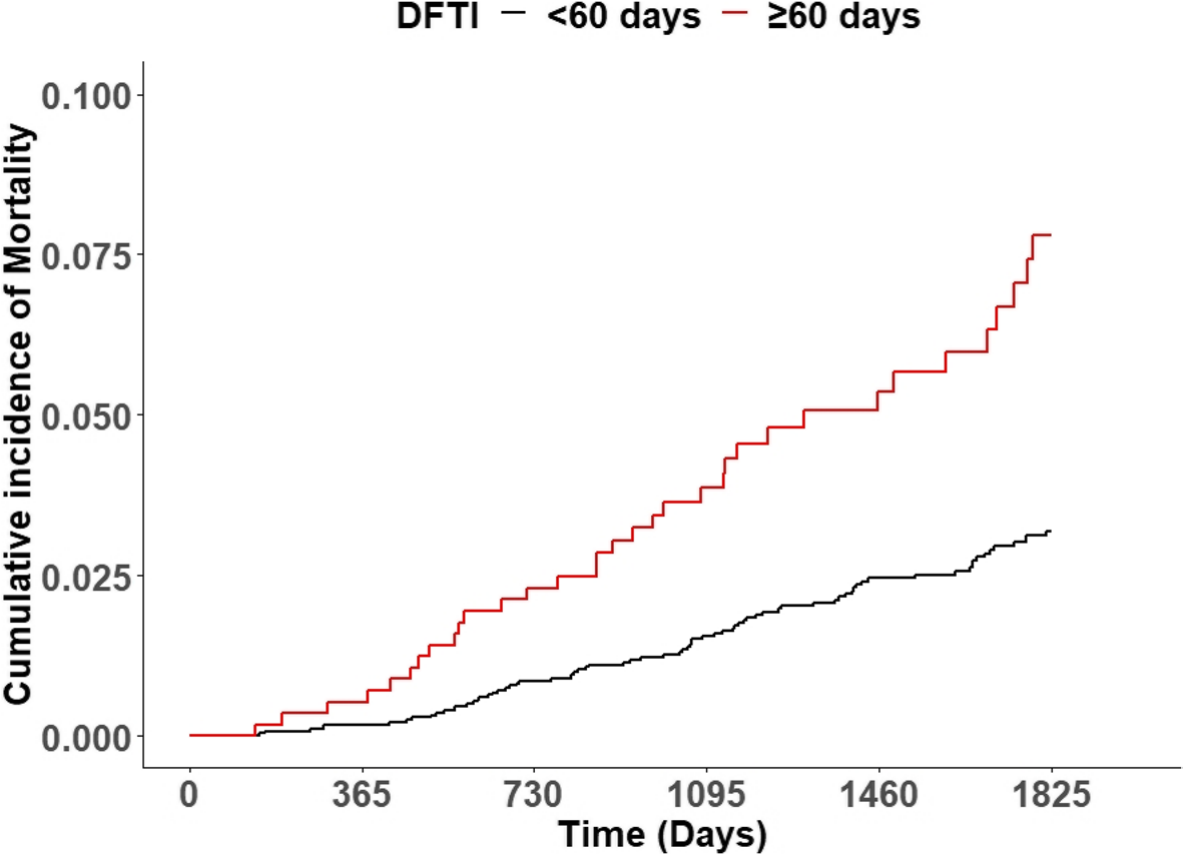
Five-year Kaplan–Meier survival curves and the results of the log-rank test. The survival rates of patients undergoing breast cancer surgery were compared, based on the diagnosis-to-first-treatment interval (DFTI) (overall period: *p* < 0.0001; log-rank test)

Figure 2 shows the Kaplan–Meier survival curves and the results of the log-rank test of the study population. The survival probability was higher in patients who received surgery within 60 days than in those who received surgery after 60 days of breast cancer diagnosis (p for log-rank test ≤ 0.0001).

Table 2 shows the results of the survival analysis using the Cox proportional hazards model that investigated the association between the DFTI and risk of mortality. Compared with the DFTI < 60 days group, the 5-year mortality rate was higher in the DFTI ≥ 60 days group (HR: 2.09, 95% CI: 1.43–3.06). Additionally, a sensitivity analysis using a DFTI threshold of 45 days revealed that, based on the Cox proportional hazards model, the 5-year mortality rate was higher in the DFTI ≥ 45 days group than in the DFTI < 45 days group (HR: 1.49, 95% CI: 1.14–1.96) (Supplementary Table 2).

**Table 2:** Cox regression analysis of DFTI and 5-year all-cause mortality in localized breast cancer

Variable	Risk of all-cause mortality	
	**HR**^a^	**95% CI**
**DFTI**		
< 60 days	**1.00**	
≥ 60 days	2.09	(1.43–3.06)
**Age (y)**		
20–54	**1.00**	
55–64	1.04	(0.61–1.76)
≥ 65	2.29	(1.46–3.59)
**Residential area**		
Urban	**1.00**	
Suburban	1.86	(1.21–2.85)
Rural	1.70	(1.11–2.58)
**Household income level**		
Low	**1.00**	
Mid-low	1.27	(0.76–2.10)
Mid-high	0.83	(0.50–1.38)
High	0.83	(0.53–1.32)
**Hospital level**		
Hospital	1.20	(0.78–1.83)
General hospital	0.74	(0.10–5.42)
Tertiary hospital	**1.00**	
**Disability**		
No	**1.00**	
Yes	2.52	(1.62–3.92)
**CCI**		
0	**1.00**	
1	1.29	(0.77–2.18)
≥ 2	1.78	(1.10–2.87)
**Treatment type**		
OP	**1.00**	
OP + CHEMO	0.32	(0.19–0.53)
OP + RADIO	0.54	(0.25–1.17)
OP + CHEMO + RADIO	0.24	(0.14–0.41)

*HR* Hazard ratio, *CI* Confidence interval, *DFTI* Diagnosis-to-first-treatment interval, *SEER* Surveillance epidemiology and end results, *CCI* Charlson comorbidity index, *OP* Operation, *CHEMO* Chemotherapy, *RADIO* Radiotherapy

^a^Adjusted for other covariates

Table 3 shows the subgroup analysis of the relationship between the DFTI and risk of mortality stratified by residential area, household income level, and the CCI. The association between the DFTI and risk of mortality was highest in patients residing in rural areas (urban, 1.28 [0.63–2.59]; suburban, 2.41 [1.20–4.83]; rural, 3.12 [1.68–5.00]), patients with low household income levels (HR [95% CI]: low, 2.99 [1.28–6.98]; mid-low, 2.12 [1.04–4.32]; mid-high, 1.92 [0.98–3.78]; and high, 1.48 [0.58–3.79]); and patients with higher CCI values (0, 1.26 [0.50–3.22]; 1, 1.35 [0.58–3.14]; ≥ 2, 2.66 [1.62–4.38]).

**Table 3:** DFTI impact on 5-year mortality varied depending on residential area, income, and CCI

Variable	Risk of all-cause mortality		
	**DFTI**		
	** < 60 days**	** ≥ 60 days**	
	**HR**	**HR**^a^	**95% CI**
**Residential area**			
Urban	1.00	1.28	(0.63–2.59)
Suburban	1.00	2.41	(1.20–4.83)
Rural	1.00	3.12	(1.68–5.80)
**Household income level**			
Low	1.00	2.99	(1.28–6.98)
Mid-low	1.00	2.12	(1.04–4.32)
Mid-high	1.00	1.92	(0.98–3.78)
High	1.00	1.48	(0.58–3.79)
**CCI**			
0	1.00	1.26	(0.50–3.22)
1	1.00	1.35	(0.58–3.14)
≥ 2	1.00	2.66	(1.62–4.38)

*HR* Hazard ratio, *CI* Confidence interval, *DFTI* Diagnosis-to-first-treatment interval, *CCI* Charlson comorbidity index

^a^Adjusted for other covariates

## Discussion

Although the importance of timely breast cancer treatment was highlighted nearly 100 years ago [48] and further reinforced by seminal research from the 1970 s [21], the impact of DFTI on breast cancer outcomes remains a persistent and significant concern in contemporary medical practice. To address this issue, our study utilized a nationally representative, population-based cohort encompassing all patients with breast cancer in Korea. After adjusting for multiple confounding factors, our findings provide valuable insights into the impact of DFTI on the risk of mortality in patients with early-stage breast cancer.

Our analysis revealed that approximately 6.1% of patients with a DFTI of ≥ 60 days experienced mortality. The risk of death was 2.09 times higher in this group than in patients with a DFTI of < 60 days. Additionally, in the sensitivity analysis, where the DFTI threshold was set at 45 days, patients with a DFTI of ≥ 45 days had a 1.49 times higher risk of mortality compared with patients with a DFTI of < 45 days. Notably, the impact of delayed treatment on mortality was particularly significant among patients from low-income households, rural areas, and patients with a high CCI.

Mortality rates after delayed treatment initiation vary significantly, ranging from 4–32% depending on factors, such as DFTI duration, follow-up period, patient race, cancer stage, and country-specific healthcare systems [19, 49]. Previous Korean studies using NHIS data primarily focused on cancers such as gastric and lung cancer [37]. To our knowledge, this study is the first in Korea to report a mortality rate of 6.1% in patients with early-stage breast cancer with a DFTI of ≥ 60 days. By comparison, a study conducted in Taiwan—a country with a healthcare system similar to that of Korea—reported a 4.5% mortality rate for patients with a DFTI of > 60 days, aligning closely with our findings [19]. Additionally, a study in China reported an 8.5% mortality rate for patients with a DFTI exceeding 14 days [50]. However, this study relied on data from a network of specific medical institutions rather than national population-based data, necessitating caution when performing direct comparisons [50]. Furthermore, research conducted in the United States reported a 5-year mortality rate of up to 11% among patients with stage 0–I breast cancer [49]. These discrepancies underscore the challenges of comparing mortality rates associated with DFTI across diverse populations and study designs. Nevertheless, our study provides meaningful insights by examining stage-specific breast cancer outcomes using data representative of the entire Korean breast cancer population.

Our findings revealed that patients with early-stage breast cancer with a DFTI of ≥ 60 days had a 2.09 -fold higher risk of mortality compared with patients with a DFTI of < 60 days. Additionally, when the DFTI threshold was adjusted to 45 days in a sensitivity analysis, the risk of death remained high, with a hazard ratio of 1.49. However, several previous studies have investigated the relationship between treatment delay and mortality risk in patients with breast cancer, yielding conflicting results. In previous research from a Korean single-center study, the association between mortality and the DFTI in patients with stage I–III breast cancer was analyzed using the cutoff values of 15, 30, 45, and 60 days; the results demonstrated that the DFTI did not affect disease-free survival [26, 27]. Furthermore, similar results were reported in studies conducted based on the DFTI cutoff values of 2 weeks, 1 month, and ≥ 2 months [25]. However, many other studies have reported results consistent with ours. Based on a domestic study, when the DFTI for patients with breast cancer was set at 1 month and 12 weeks, the 5-year mortality rate was 1.59 times higher [17], and when the DFTI was set at 12 weeks, it was 1.91 times higher [51]. In addition, based on the results of a study conducted on the Taiwanese population, employing a similar study design as ours, the risk of death was 2.93 times higher when the DFTI was ≥ 3 months in patients with stage II breast cancer [19]. In addition, a study examining the 60-day DFTI reported that the risk of death was highest in patients with stage I breast cancer at 1.13 times, followed by those with stages II breast cancer at 1.06 times; however, no statistically significant value was found in patients with stage III breast cancer [10]. These discrepancies in study findings can be attributed to differences in the study population, cancer stage, time frames, and study design.

As observed in our study, the relationship between delayed surgical treatment and high 5-year mortality in patients with early-stage breast cancer can be partly attributed to the fact that timely resection has a key role in improving overall survival in early-stage breast cancer. This effect of the DFTI and mortality, especially in patients with early-stage breast cancer, can be explained by the theory proposed by Bleicher et al. [10]. Patients with high breast cancer stage already exhibit a high underlying mortality rate, and those with early-stage breast cancer have a relatively low underlying mortality rate, making them more affected by the DFTI [10]. In patients with late-stage breast cancer, the outcome of cancer is biologically determined, and small effects of reduction of the DFTI in days or months have no significant impact on mortality [10]. Thus, the overall survival of patients with early-stage breast cancer is better than that of patients with late-stage breast cancer, suggesting that the relative impact of treatment delays can have a significant impact on prognosis. Based on these theories and previous findings, the DFTI in patients with early-stage breast cancer may be even more important, and our results analyzing the relationship between the DFTI and mortality in patients with early-stage breast cancer using a large national population-based cohort in South Korea are noteworthy. Additionally, our study adds evidence to the recommendations of the National Breast and Cervical Cancer Early Detection Program [52] that longer waiting times for treatment may have a greater impact on mortality, and that delayed treatment may increase a patient's risk of cancer progression [52]. Delaying surgical treatment may exacerbate patients'anxiety and lead to psychiatric complications, further contributing to adverse effects, such as postponement of adjuvant treatment [14, 15]. As the preoperative stage is a risk factor for predictive survival, timely treatment may constitute an important factor, regardless of age [53]. This suggests that efforts to shorten the DFTI in patients with early-stage breast cancer may be an important factor in improving the quality of breast cancer treatment and highlights the need for policies focused on monitoring appropriate and timely treatments at the system level.

Our subgroup analysis revealed that the negative impact of the DFTI was particularly pronounced among patients residing in rural areas, those with low household income levels, and those with higher CCI. The negative impact of DFTI resulting from low socioeconomic status, such as low household income and residence in rural areas, could be interpreted in terms of disparities in healthcare resources and accessibility [54]. In Korea, the unequal distribution of medical facilities, state-of-the-art treatment facilities, and diverse specialized personnel between urban and rural areas pose significant concerns [55]. The well-equipped medical facilities and physician-to-patient ratios in capital areas may lead to better patient outcomes, ultimately impacting mortality rates [56]. Furthermore, individuals with low-income levels may encounter barriers in accessing large-scale tertiary medical institutions, potentially restricting timely and consistent follow-up care and monitoring [57, 58]. Moreover, considering the limited time available for medical diagnosis and treatment coupled with the fewer treatment options available compared with higher income groups [19], treatment delays could have had a greater impact on mortality rates among the low-income population. Furthermore, our results show that the negative impact of the DFTI increases with higher CCI, potentially due to reduced expectations of treatment benefits and the challenges of managing comorbidities [59]. Previous studies have highlighted that high CCI is associated with poor treatment resistance and the necessity of evaluating functional status and comorbidities before determining optimal treatment methods [60, 61]. Therefore, a higher CCI may have influenced subsequent treatments and, in turn, amplified the impact of DFTI on mortality risk in patients with early-stage breast cancer.

This study has some limitations. First, this was a retrospective analysis, and we could not accurately evaluate the cause of delayed treatment. Similarly, the interval from symptom expression to diagnosis remains unknown. Second, the disease codes used in the inclusion criteria may not match the actual disease status of the patient, which is a fundamental limitation of the insurance database [62]. However, almost all hospitals in South Korea operate under the reimbursement system, and all surgical procedures and treatments are recorded in the claims database. Furthermore, based on a similar previous study that reported the sensitivity of identifying patients with cancer by comparing National Health Insurance claims data and central registry claim data, the sensitivity for breast cancer was 98.1% [31]. Based on the results of the above studies, the accuracy of breast cancer diagnosis in this study is considered highly reliable. Third, because we used retrospective cohort data, we were unable to incorporate certain potential covariates that influence breast cancer mortality, such as education level, household size, family history of breast cancer, health literacy, hormone receptor status, and tumor characteristics, including triple-negative status and other prognostic indicators (e.g., tumor grade, HER2 status). This limitation is due to the nature of the claims data used in our study. However, the data did include information on disability status, comorbidities, and types of treatment. Furthermore, other studies conducted by integrating KNCI DB with NHIS data have selected participants by considering cancer stages, which are often overlooked [63, 64]. Fourth, the primary objective of this study was to examine the effect of DFTI on mortality. Thus, although treatment type was included as a covariate, the specific order of treatments, such as whether chemotherapy was administered preoperatively or postoperatively, was not considered, despite its emphasis in previous studies [65]. Nonetheless, treatment types were tracked for up to 1 year after the diagnosis and incorporated as a covariate in the analysis of the association between DFTI and mortality. Future research should aim to account for the sequence of treatment types to provide a more comprehensive understanding of these relationships. Fifth, selection and unmeasured bias may exist. However, to reduce these biases as much as possible and increase sample homogeneity, patients included in this study were limited to those with new-onset breast cancer, and PSM and strict exclusion criteria were applied. Sixth, in the current study, 8,080 of the 67,510 included patients (11.96%) were excluded because of the stringent exclusion criteria. However, only 128 patients (0.1%) were excluded due to missing data on other covariates. Considering that this study encompassed all breast cancer cases in Korea, the exclusion of 128 patients due to missing data was considered negligible and unlikely to impact the overall findings. Finally, a causal relationship could not be established because of the retrospective nature of the data. The increased mortality risk due to delayed treatment may be a temporary relationship rather than a causal relationship.

Despite these limitations, our study has certain strengths. To the best of our knowledge, this is the first study to analyze national-level data to investigate the relationship between the DFTI and mortality, focusing on Korean patients with early-stage breast cancer. Additionally, unlike previous studies, this study was conducted using a nationwide database and was not limited to specific hospitals or institutions [26, 27]. Therefore, the results of this study could be generalized to individuals with early-stage breast cancer in Korea with similar demographic characteristics or to the same patient cohort in other countries and could provide a background for managing the timing of treatment after diagnosis to reduce mortality in this patients population. In this study, PSM was applied to the exposure variables to reduce the heterogeneity between the DFTI < 60 days and DFTI ≥ 60 days groups. Furthermore, only patients with new-onset breast cancer were included, and the effect of immortal time bias was also considered [29]. Therefore, confounding factors may have been reduced and the comparability may have been improved between the patients in the DFTI ≥ 60 days and DFTI ≥ 60 days groups.

## Conclusions

A DFTI ≥ 60 days is associated with mortality risk in patients with early-stage breast cancer. Patients with a DFTI ≥ 60 days had a 2.09 times higher 5-year mortality risk. Additionally, this risk was stronger in patients from low-income households, rural residents, and those with high CCI. These findings confirm the importance of systems that monitor the DFTI to reduce the risk of death in patients with early-stage breast cancer with low baseline mortality rates. Moreover, this indicates that improving breast cancer treatment outcomes does not depend solely on early diagnosis as has been traditionally perceived but also on post-diagnosis actions.

## Supplementary Information

Supplementary Material 1.

Supplementary Material 2.

## Data Availability

No datasets were generated or analysed during the current study.

## Abbreviations

DFTI: Diagnosis-to-First-Treatment Interval
NHIS: National Health Insurance Service
KCCR: Korea Central Cancer Registry
KNCI DB: Korea National Cancer Incidence Database
SEER: Surveillance, Epidemiology, and End Results
ICD-10: International Statistical Classification of Diseases and Related Health Problems, Tenth Revision
PSM: Propensity Score Matching
HR: Hazard Ratio
CI: Confidence Interval
CCI: Charlson Comorbidity Index
