# Supplementary material for: Impact of diagnosis-to-treatment interval on mortality in patients with early-stage breast cancer: a retrospective nationwide Korean cohort
Source: BMC Womens Health. 2025 May 22;25:247. doi: 10.1186/s12905-025-03780-6 (PMC12096538; doi:10.1186/s12905-025-03780-6)
Supplement: Supplementary file 1 — Supplementary Material 1. [file 12905_2025_3780_MOESM1_ESM.docx]

**Supplementary Table 1. Distribution of all-cause mortality based on DFTI and other covariates**

| **Variable** | **Total** | | **Risk of all-cause mortality** | | | | **p-value** |
| --- | --- | --- | --- | --- | --- | --- | --- |
|  |  |  | **No** | | **Yes** | |  |
| **Total** | **4350** | **(100.0)** | **4219** | **(97.0)** | **131** | **(3.0)** |  |
| **DFTI** |  |  |  |  |  |  | <.0001 |
| <60 days | 3625 | (83.3) | 3538 | (97.6) | 87 | (2.4) |  |
| ≥60 days | 725 | (16.7) | 681 | (93.9) | 44 | (6.1) |  |
| **Age (y)** |  |  |  |  |  |  | <.0001 |
| 20–54 | 2526 | (58.1) | 2478 | (98.1) | 48 | (1.9) |  |
| 55–64 | 978 | (22.5) | 957 | (97.9) | 21 | (2.1) |  |
| ≥65 | 846 | (19.4) | 784 | (92.7) | 62 | (7.3) |  |
| **Region** | 0 |  |  |  |  |  | 0.0019 |
| Urban | 2256 | (51.9) | 2208 | (97.9) | 48 | (2.1) |  |
| Suburban | 996 | (22.9) | 957 | (96.1) | 39 | (3.9) |  |
| Rural | 1098 | (25.2) | 1054 | (96.0) | 44 | (4.0) |  |
| **Household income level** |  |  |  |  |  |  | 0.2252 |
| Low | 1086 | (25.0) | 1044 | (96.1) | 42 | (3.9) |  |
| Mid-low | 822 | (18.9) | 796 | (96.8) | 26 | (3.2) |  |
| Mid-high | 1026 | (23.6) | 1000 | (97.5) | 26 | (2.5) |  |
| High | 1416 | (32.6) | 1379 | (97.4) | 37 | (2.6) |  |
| **Disability** |  |  |  |  |  |  | <.0001 |
| No | 4087 | (94.0) | 100 | (2.4) | 3987 | (97.6) |  |
| Yes | 263 | (6.0) | 31 | (11.8) | 232 | (88.2) |  |
| **Hospital level** |  |  |  |  |  |  | 0.2056 |
| Hospital | 48 | (1.1) | 1 | (2.1) | 47 | (97.9) |  |
| General hospital | 690 | (15.9) | 28 | (4.1) | 662 | (95.9) |  |
| Tertiary hospital | 3612 | (83.0) | 102 | (2.8) | 3510 | (97.2) |  |
| **Charlson Comorbidity Index** |  |  |  |  |  |  | <.0001 |
| 0 | 1582 | (36.4) | 28 | (1.8) | 1554 | (98.2) |  |
| 1 | 1254 | (28.8) | 30 | (2.4) | 1224 | (97.6) |  |
| ≥2 | 1514 | (34.8) | 73 | (4.8) | 1441 | (95.2) |  |
| **Treatment type** |  |  |  |  |  |  | <.0001 |
| OP | 187 | (4.3) | 26 | (13.9) | 161 | (86.1) |  |
| OP+CHEMO | 1210 | (27.8) | 44 | (3.6) | 1166 | (96.4) |  |
| OP+RADIO | 167 | (3.8) | 9 | (5.4) | 158 | (94.6) |  |
| OP+CHEMO+RADIO | 2786 | (64.0) | 52 | (1.9) | 2734 | (98.1) |  |

DFTI, diagnosis-to-first-treatment interval; OP, operation; CHEMO, chemotherapy; RADIO, radiotherapy
