# Supplementary material for: Impact of diagnosis-to-treatment interval on mortality in patients with early-stage breast cancer: a retrospective nationwide Korean cohort
Source: BMC Womens Health. 2025 May 22;25:247. doi: 10.1186/s12905-025-03780-6 (PMC12096538; doi:10.1186/s12905-025-03780-6)
Supplement: Supplementary file 2 — Supplementary Material 2. [file 12905_2025_3780_MOESM2_ESM.docx]

**Supplementary Table 2. Sensitivity analysis results of DFTI (45-day cutoff) and 5-year all-cause mortality in localized breast cancer**

| **Variables** | **After PSM** | | | | | | | | | | |
| --- | --- | --- | --- | --- | --- | --- | --- | --- | --- | --- | --- |
|  | **Total** | | **DFTI** | | | | **p-value** | **Risk of all-cause mortality** | | | |
|  |  |  | **≥45 days** | | **<45 days** | |  |  |  |  |  |
| **Total** | **10,356** | **(100.0)** | **1726** | **(16.7)** | **8630** | **(83.3)** |  | **HR^a^** | **95% CI** | | |
| **DFTI** |  |  |  |  |  |  |  |  |  |  |  |
| <45 days | 8630 | (83.3) |  |  |  |  |  | 1.00 |  |  |  |
| ≥45 days | 1726 | (16.7) |  |  |  |  |  | 1.49 | (1.14 | - | 1.96) |
| **Age (years)** |  |  |  |  |  |  | 1.0000 |  |  |  |  |
| 20–54 | 6234 | (60.2) | 1039 | (16.7) | 5195 | (83.3) |  | 1.00 |  |  |  |
| 55–64 | 2292 | (22.1) | 382 | (16.7) | 1910 | (83.3) |  | 1.32 | (0.95 | - | 1.83) |
| ≥65 | 1830 | (17.7) | 305 | (16.7) | 1525 | (83.3) |  | 2.59 | (1.93 | - | 3.48) |
| **Region** |  |  |  |  |  |  | 1.0000 |  |  |  |  |
| Urban | 4992 | (48.2) | 832 | (16.7) | 4160 | (83.3) |  | 1.00 |  |  |  |
| Suburban | 2388 | (23.1) | 398 | (16.7) | 1990 | (83.3) |  | 1.06 | (0.78 | - | 1.44) |
| Rural | 2976 | (28.7) | 496 | (16.7) | 2480 | (83.3) |  | 1.18 | (0.91 | - | 1.54) |
| **Household income level** |  |  |  |  |  |  | 1.0000 |  |  |  |  |
| Low | 2496 | (24.1) | 416 | (16.7) | 2080 | (83.3) |  | 1.00 |  |  |  |
| Mid-low | 1782 | (17.2) | 297 | (16.7) | 1485 | (83.3) |  | 0.60 | (0.42 | - | 0.88) |
| Mid-high | 2424 | (23.4) | 404 | (16.7) | 2020 | (83.3) |  | 0.69 | (0.51 | - | 0.94) |
| High | 3654 | (35.3) | 609 | (16.7) | 3045 | (83.3) |  | 0.55 | (0.41 | - | 0.73) |
| **Hospital level** |  |  |  |  |  |  | 1.0000 |  |  |  |  |
| Hospital | 66 | (0.6) | 11 | (16.7) | 55 | (83.3) |  | 1.19 | (0.89 | - | 1.59) |
| General hospital | 1356 | (13.1) | 226 | (16.7) | 1130 | (83.3) |  | 0.44 | (0.06 | - | 3.12) |
| Tertiary hospital | 8934 | (86.3) | 1489 | (16.7) | 7445 | (83.3) |  | 1.00 |  |  |  |
| **Disability** |  |  |  |  |  |  | 0.0835 |  |  |  |  |
| No | 9797 | (94.6) | 1618 | (16.5) | 8179 | (83.5) |  | 1.00 |  |  |  |
| Yes | 559 | (5.4) | 108 | (19.3) | 451 | (80.7) |  | 2.20 | (1.61 | - | 3.01) |
| **CCI** |  |  |  |  |  |  | 0.6391 |  |  |  |  |
| 0 | 3683 | (35.6) | 631 | (17.1) | 3052 | (82.9) |  | 1.00 |  |  |  |
| 1 | 3074 | (29.7) | 505 | (16.4) | 2569 | (83.6) |  | 1.22 | (0.88 | - | 1.70) |
| ≥2 | 3599 | (34.8) | 590 | (16.4) | 3009 | (83.6) |  | 1.35 | (1.00 | - | 1.84) |
| **Treatment type** |  |  |  |  |  |  | <.0001 |  |  |  |  |
| OP | 434 | (4.2) | 130 | (30.0) | 304 | (70.0) |  | 1.00 |  |  |  |
| OP+CHEMO | 2816 | (27.2) | 623 | (22.1) | 2193 | (77.9) |  | 0.57 | (0.40 | - | 0.82) |
| OP+RADIO | 298 | (2.9) | 81 | (27.2) | 217 | (72.8) |  | 0.68 | (0.38 | - | 1.24) |
| OP+CHEMO+RADIO | 6808 | (65.7) | 892 | (13.1) | 5916 | (86.9) |  | 0.30 | (0.21 | - | 0.44) |

HR, hazard ratio; CI, confidence interval; DFTI, diagnosis-to-first-treatment interval; SEER, surveillance epidemiology and end results; CCI, Charlson comorbidity index; OP, operation; CHEMO, chemotherapy; RADIO, radiotherapy.

^a^ Adjusted for other covariates
